# Connective tissue disease with macrophage activation syndrome: A case report

**DOI:** 10.1097/MD.0000000000032426

**Published:** 2022-12-23

**Authors:** Qu Chen, Qiushuang Zhang, Xuebin Wang

**Affiliations:** a Department of Rheumatology, Binzhou Medical University Hospital, Binzhou, China; b Department of Rheumatology, Binzhou Medical University Hospital, Binzhou, China.

**Keywords:** case report, hemophagocytic lymphocytosis, hemophagocytic syndrome, macrophage activation syndrome, tocilizumab

## Abstract

Macrophage activation syndrome (MAS), or secondary hemophagocytic lymphocytosis (sHLH), is a rare systemic inflammatory response syndrome that is fatal. Adult patients lack clear criteria for diagnosis and treatment, primarily derived from guidelines and protocols for treating family hemophagocytic lymphocytosis and systemic juvenile idiopathic arthritis (sJIA)-related MAS in children or from retrospective case reports. As a subtype of sHLH, MAS has a clinical presentation like sHLH, but treatment varies. Herein, we report the case of a 40-year-old female with MAS caused by a connective tissue disease.

**Patient concerns:**

The patient presented to the Rheumatology and Immunology Clinic with recurrent fever and rash, and MAS was confirmed after a series of examinations. The patient had no significant effect after treatment with JAK inhibitors, but after the use of the IL-6 inhibitor tocilizumab, the fever and rash were significantly reduced, and laboratory indicators returned to normal levels.

**Diagnosis:**

Considering the patient’s condition and laboratory test results, we judged that the patient had connective tissue disease with MAS.

**Interventions:**

We gave sequential treatment of tocilizumab.

**Outcomes:**

ALL indicators are mostly back to normal when the patient was monitored at the outpatient clinic.

**Lessons:**

MAS/HLH lacks clear criteria for diagnosis or treatment in adult patients and is extremely difficult to distinguish from bacterial sepsis or other systemic inflammatory response syndromes. Consequently, early diagnosis and treatment are indispensable for enhancing patient survival.

## 1. Introduction

Hemophagocytic lymphocytosis/macrophage activation syndrome (HLH/MAS) is a group of acute systemic inflammatory response syndromes caused by abnormal activation of cytotoxic lymphocytes and macrophages, which secrete many pro-inflammatory cytokines such as interferon γ (IFN-γ), tumor necrosis factor α (TNF-α), and interleukin (IL)-1, IL-4, IL-6, IL-8, IL-10, and IL-18. It is also known as cytokine storm syndrome.^[1]^ IL-6 can reduce the cytolytic function of NK cells, prolong the interaction between innate and adaptive immune cells, and amplify the pro-inflammatory cytokine cascade of MAS.^[2]^

According to previous studies, nearly all rheumatic diseases have been reported as secondary MAS, but the most prevalent are systemic juvenile idiopathic arthritis (sJIA), adult onset Still's disease, systemic lupus erythematosus, Kawasaki disease.^[3]^The overall mortality rate for adult HLH patients is high, ranging between 40% to 70%.^[4]^In addition, within two months of diagnosis, more than 10% of patients with HLH die from internal organ bleeding, neutropenia, or opportunistic infections caused by Multiple organ failure. Therefore, prompt diagnosis and treatment are required for HLH/MAS. We reported patient with connective tissue disease and MAS, whose main manifestations were “fever and rash.” The fever was effectively controlled after immunosuppressive therapy with tocilizumab. The specific diagnose and treatments were as follows:

## 2. Case report

**Figure 1.**
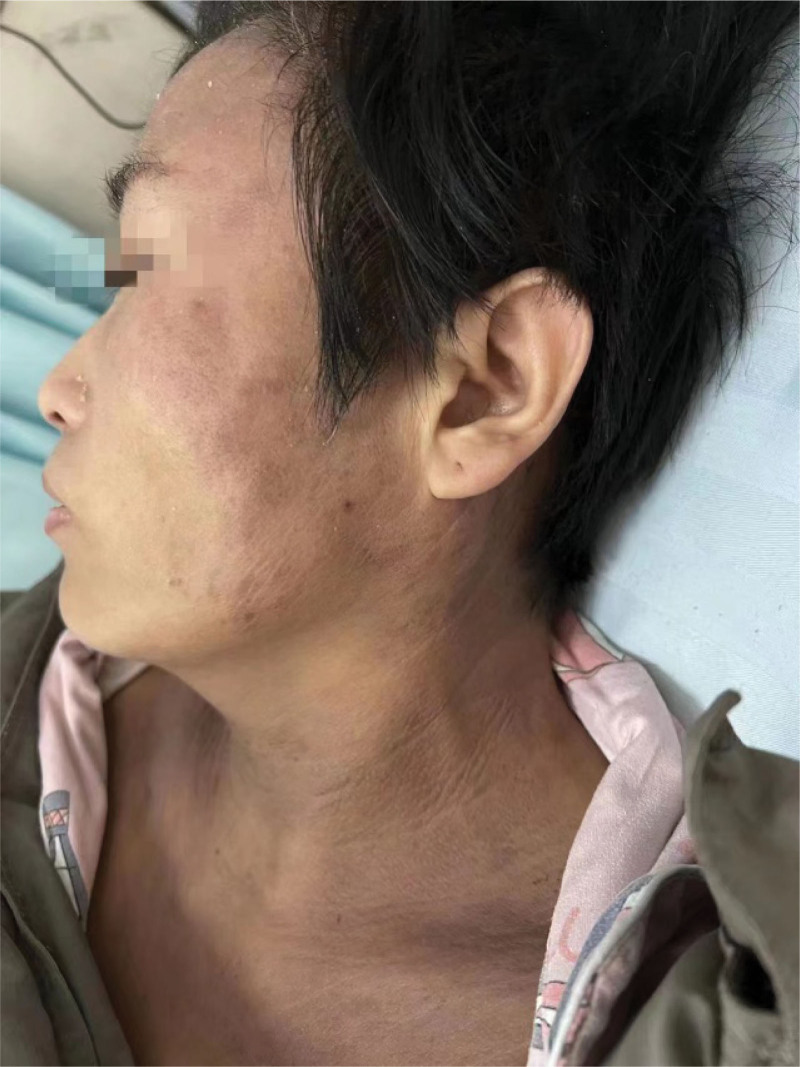
The purple-red rash in the V-shaped area of the hairline, neck, and front chest of patient.

**Figure 2.**
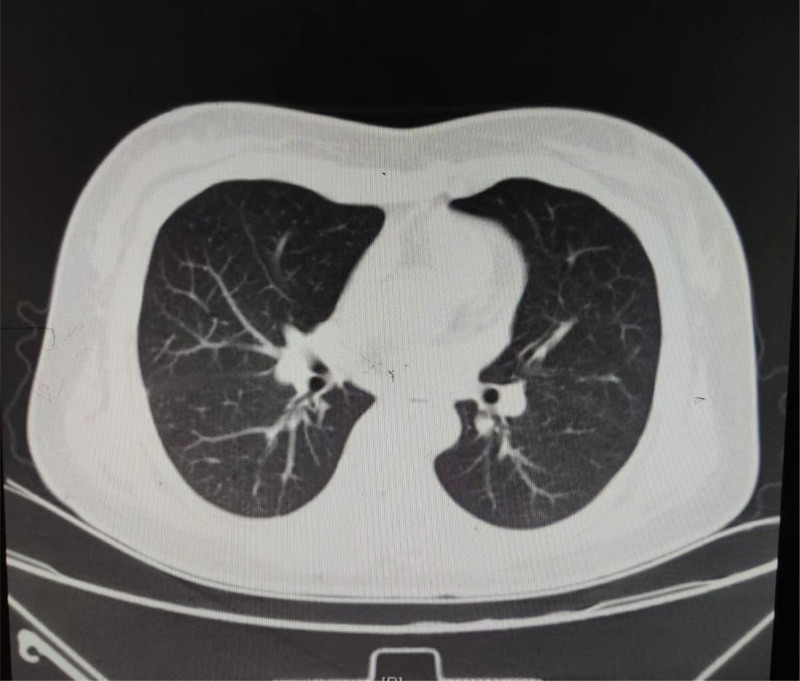
The chest CT of patient.

A year ago, the patient had a fever with no apparent precipitating factors; a body temperature of up to 39.7°C, a purple-red rash in the V-shaped area of the hairline, neck and front chest; and general body weakness (Fig. 1). Therefore, she visited the local hospital for a battery of tests and found that there was abnormal liver function and elevated lactate dehydrogenase (Table 1), with ANA 1: 80(+) and, SMA (a anti-smooth muscle antibodies) ± PETCT: Increased FDG metabolism in splenomegaly, polyoskeletal, and multi-region lymph nodes. Lymph node pathological biopsy (cervical lymph nodes): proliferative lesions of lymphoid tissue. Cutaneous pathology suggests chronic dermatoid changes. After administering Ebastin tablets, halometasone cream, and other treatments, the rash improved slightly, but the fever persisted, and the patient’s body temperature reached a maximum of 39°C. The patient was admitted to our hospital on December 4, 2021, exhibiting a purple-red rash in the V region of the hairline, neck, and anterior chest, along with itching and pigmentation. Laboratory tests reveal elevated white blood cells, erythrocytes, C-reactive protein, liver enzymes, and ferritin (Table 1), ANA 1: 320(+). In addition, cytokine detection revealed a significant increase in the IL-6. And the chest CT: Bilateral axillary lymph nodes are slightly larger and the spleen is larger (Fig. 2). We believed the patient had “connective tissue disease dermatomyositis” because the rash improved, and the patient was discharged following treatment with Methylprednisolone 40 mg/qd, Methotrexate, and Baricitinib. The patient had recurrent fever after discontinuation of the hormone outside the hospital, and laboratory tests found abnormal sCD25 and NK cell activity, in addition to elevated leukocytes, liver enzymes, and ferritin (Table 1). The patient was discharged from the hospital with an improved body temperature after receiving ruxolitinib and hormone therapy for macrophage activation syndrome. The patient was administered oral “prednisone acetate tablets and ruxolitinib,” and the fever returned when the hormone was gradually reduced to 5 mg/d. The patient’s body temperature peaked at 39.8°C, and the rash worsened.

**Table 1:** The change in clinical and laboratory manifestations.

	February 2021	December 2021	January 2022	February 2022	May 2022
Temperature (℃)	39.7	38.9	39.8	38.5	36.8
WBC (4–10 × 10^9^/L)	5.73	14.0	14.08	20.1	9.2
Hb (110–150 g/L)	108	102	99	103	118
Plt (100–300 × 10^9^/L)	389	417	419	431	352
RBC (3.5–5.5 × 10^12^/L)	3.8	3.6	3.9	4.0	4.5
AST (15–40 U/L)	155	68.8	50.8	81.10	46.6
ALT (9–50 g/L)	30.4	100.6	17	88.40	22.6
LDH (120–250 U/L)	1490	430	539	390.10	248
Ferritin (13–150 g/mL)	–	1972	966.4	1035	233.2
sCD25 (<6400 pg/mL)	–	–	8151	–	–
NK-cell activity (47.6–76.8%)	–	–	12.25	–	–
Fibrinogen (2–4 g/L)	–	5.5	5.1	4.8	2.1
Dimer (0–0.5 mg/L)	–	1.58	2.27	4.97	–
ESR (0–20 mm/h)	–	74	90	120	50
CRP (0–6 mg/L)	–	54.2	56.9	48.7	41.5
Cytokines					
IL-2 (1–5.71 pg/mL)	–	1.67	–	–	0.86
IL-4 (0–2.8 pg/mL)	–	4.63	–	–	1.43
IL-6 (0–7 pg/mL)	–	150.29	–	–	237.33
IL-10 (0–4.91 pg/mL)	–	6.02	–	–	1.41
TNF-α (0–2.31 pg/mL)	–	1.37	–	–	0.89
IFN-γ (0–7.42 pg/ml)	–	3.91	–	–	2.71

ALT = alanine aminotransferase, AST = aspartate aminotransferase, CRP = C-reactive protein, ESR = erythrocyte sedimentation), Hb = hemoglobin, LDH = lactate dehydrogenase, Plt = platelet, RBC = red blood cell, WBC = white blood cell.

Owing to the patient’s long-term use of hormones and immunosuppressants, it was impossible to rule out the possibility of infection. Therefore, we added anti-infective moxifloxacin and anti viral ganciclovir therapy. In addition, 5 mg of anti-inflammatory prednisone acetate was continued to treat connective tissue disease with the addition of hydroxychloroquine. Unfortunately, the patient continued to experience recurrent high fever, the rash remained unchanged, and the antibiotics were ineffective. Considering the patient’s condition, we judged that the patient had connective tissue disease with MAS. JAK inhibitors poorly controlled the patient’s disease. Therefore, on March 2, 2022, we discontinued JAK inhibitors and added tocilizumab immunosuppressive therapy. The patient did not have fever, and the rash gradually disappeared. After being released from the hospital, the patient routinely took the following medications: “prednisone acetate tablets 5 mg/qd, hydroxychloroquine sulfate 0.2 g bid.” All indicators mostly returned to normal when the patient was monitored at the outpatient clinic on May 25, 2022.

## 3. Discussion

Persistent hyperthermia, rash, hepatosplenomegaly, pancytopenia, elevated liver enzyme and ferritin levels, and coagulation dysfunction are vital clinical characteristics of HLH/MAS.^[5]^ Most information regarding the diagnosis and treatment of HLH comes from the pediatric literature, with the HLH-2004 diagnostic and treatment criteria being the most prevalent. The diagnosis of HLH is determined when more than five of these eight items are met: fever; splenomegaly; cytopenia affecting more than two cells in three cell lines; hypertriglyceridemia or hypofibrinogenemia; finding hemophagocytophagocytes in bone marrow, spleen, or lymph nodes; hypoactivity or deletion of NK cells; hyperferritinemia; elevated levels of sIL-2R or sCD25.^[6]^ In addition to these criteria, diagnostic MAS includes MAS/sJIA standards designed for MAS and sJIA mergers.^[7]^ However, such criteria are derived from observations of pediatric patients and have not been validated in adult patients. Therefore, they have certain limitations. Some hospitals cannot measure NK cell activity and soluble IL-2 receptors following the HLH-2004 standard. Missed or incorrect diagnose frequently occur when patients in the early stages of a disease do not exhibit clinical symptoms.

In this case report, the patient primarily presented with recurrent fever and rashes. Admission examination revealed a purple-red rash on the hairline, neck, and anterior chest V-zone, accompanied by itching and pigmentation, laboratory tests suggesting ana-positivity, and a precise diagnosis of connective tissue disease. In addition, the patient’s purple-red rash at the hairline, neck, and V area of the anterior chest did not exclude the possibility of dermatomyositis, so anti-inflammatory and immunosuppressive therapy with “Methylprednisolone, Methotrexate, and JAK inhibitor Baricitinib” was administered. Upon further examination, the patient was determined to have fever, splenomegaly, hyperferritinemia, decreased NK cell activity, and elevated sCD25 levels. Therefore, according to the HLH-2004 diagnostic criteria, the patient was diagnosed with HLH, and MAS was caused by connective tissue disease.

Most treatments for adults with HHLH/MAS are based on guidelines and protocols for treating pediatric HLH, sJIA-related MAS, or retrospective case reports.^[8]^ The treatment of HLH in children is based on HLH-1994 or HLH-2004 regimens (etoposide, dexamethasone, cyclosporine A, and, in some patients, intrathecal methotrexate), and chemotherapy combined with immunotherapy effectively controls the disease in most patients. After drug therapy, allogeneic hematopoietic stem cell transplantation is necessary for patients with familial, severe, persistent, or recurrent HLH. Adults with HLH can benefit from the HLH-2004 protocol; however, the dosage and medication type must be modified. In addition, some experts believe that chemotherapy drugs, such as etoposide, are ineffective in treating MAS and should be administered with caution.^[9]^

The basic HLH regimen for these children lacks a quantifiable dose or toxicity when applied to adults. Immunosuppressants have been prominently used in the treatment of HLH/MAS over the past decade. IL-1, IL-6, IL-18, JAK, and IFN-γ antagonists have emerged following the pathogenesis of HLH/MAS. Studies have demonstrated that the JAK inhibitor ruxolitinib can alleviate the symptoms of secondary HLH in adults.^[4]^According to Rebecca et al,^[10]^ alemtuzumab, a CD52 antibody against T cell and tissue cell expression, was effective in treating refractory HLH in children and adults. In addition, the IL-1 inhibitor anakinra has been associated with a reduction in C-reactive protein and ferritin levels in patients with MAS diagnosed with sJIA.^[11]^ Masaki et al found that compared to untreated patients with sJIA-associated MAS, patients treated with tocilizumab-treated sJIA-associated MAS had significantly reduced fever and levels of ferritin, triglycerides, and C-reactive protein. Other laboratory features of MAS, such as decreased platelet counts and fibrinogen levels are more pronounced in patients treated with tocilizumab.^[12]^ Barsalou et al^[13]^ recently reported effective treatment of HLH in neonates with NLRC4 functional mutations by combining anakinra and rapamycin. A study found that rapamycin decreased macrophage secretion of IL-18 and IL-1β by reducing caspase-1 activation, indicating that inhibition of mTOR may be advantageous for patients with inherited autoimmune diseases. IL-1 blockade appears to be more effective than IL-6 blockade in treating MAS, and intravenous immunoglobulins (IVIg) should be considered when IL-1 inhibitors are ineffective.^[5]^ HLH/MAS is also treated with tacrolimus, rituximab, and plasmapheresis, in addition to the drugs listed above.^[14]^

After confirming the MAS diagnosis, we initially administered a JAK inhibitor and hormone therapy, but the patient’s fever did not improve significantly. According to the patient’s cytokine test results, the patient had an elevated IL-6 level; therefore, we attempted to apply the IL-6 inhibitor tocilizumab sequential therapy, and the patient’s fever was effectively controlled. At the same time, liver function and ferritin levels were significantly lower than before, which was consistent with the results of Masaki et al in the treatment of sJIA-related MAS patients with tocilizumab.

## 4. Conclusions

HLH/MAS manifests clinically similar to bacterial sepsis or systemic inflammatory response syndrome, with recurrent fever and ineffective antibiotic treatment. Before initiating treatment, clinicians should not wait for patients’ symptoms and laboratory tests to fully meet the HLH-2004 or MAS/sJIA diagnostic criteria. When patients present with unexplained fever accompanied by impaired multi-organ function, cytopenia, or hyperferritinemia, HLH/MAS may be suspected, and relevant laboratory tests should be improved to avoid delaying HLH/MAS treatment. In addition, antagonists of IFN-γ, IL-1, IL-6, JAK, IL-18, and other factors play a significant role in pathogenesis of MAS/HLH. Moreover, antagonists should be selected based on various precipitation factors and populations. Tocilizumab is an anti-IL-6 agent. The effect of the sJIA/MAS treatment was remarkable. In this case, JAK inhibitors were ineffective. Tocilizumab not only improves fever and rash, but also improves ferritin levels and liver function. Therefore, treatment can be attempted when a patient’s fever cannot be controlled effectively. Future clinical trials must determine more suitable treatments for adult patients with MAS/HLH, and the efficacy of mTOR and IL-18 inhibitors must be investigated in greater depth.

## Author contributions

CQ wrote the case report, ZQS helped revise the manuscript, WXB helped choose the patient, and ZQS, WXB treated this patient. All authors read and approved the final manuscript.

**Funding acquisition:** Xuebin Wang.

**Investigation:** Qu Chen, Qiushuang Zhang.

**Resources:** Qiushuang Zhang.

**Supervision:** Qiushuang Zhang.

**Writing – original draft:** Qu Chen.

**Writing – review & editing:** Xuebin Wang.
